# A Review of High-Intensity Focused Ultrasound in Urology

**DOI:** 10.3390/cancers13225696

**Published:** 2021-11-14

**Authors:** David Cranston, Tom Leslie, Gail ter Haar

**Affiliations:** 1 Nuffield Department of Surgery, University of Oxford, Oxford OX3 9DU, UK; 2 Oxford University Hospitals Foundation Trust, Oxford OX3 7LE, UK; tom.leslie@ouh.nhs.uk; 3 Department of Physics, The Institute of Cancer Research, London SW7 3RP, UK; Gail.terHaar@icr.ac.uk

**Keywords:** high-intensity focused ultrasound, kidney cancer, prostate, prostatic carcinoma

## Abstract

**Simple Summary:**

High intensity focused ultrasound is a novel and non invasive treatment for an increasing number of cancers and benign diseases. The principal is similar to focusing the sun’s rays with a magnifying glass, causing a burn at the focal point. This can be done with ultrasound if the usual diagnostic energy is increased 10,000 times. The ultrasound energy is generated under water and the energy passed noninvasively into the body to a sharp focus where it destroys tissue. The technology is rapidly evolving. In urology, trials have been done in a number of areas but the two which are most promising at the present time are for the treatment of renal and prostate cancers. Treatment of renal cancer is currently limited by the position of the kidneys due to the ribs and the presence of perinephric fat, but treatment for prostate cancer is becoming more established.

**Abstract:**

This review provides an introduction to high-intensity focused ultrasound (HIFU) and reviews its historical and current use in urological surgery. Current and historical literature (1927–2020), including that describing trials and review articles in the medical and ultrasonic literature, has been reviewed, using Pub Med and Cochrane search engines. HIFU is currently one of a number of treatments for prostate cancer, both as a primary treatment that can be repeated, and as a salvage treatment post-radiotherapy. HIFU is not yet sufficiently mature to be a standard treatment for renal cancer or other urological diseases, although there has been some success in early clinical trials. As the technology improves, this situation is likely to change. HIFU has been understood as a concept for a century, and has been applied in experimental use for half that time. It is now an accepted treatment with low morbidity in many diseases outside the scope of this review. In urological surgery, prostate HIFU is accepted as a localised treatment in selected cases, with potentially fewer side effects than other localised therapies. Currently the treatment for renal cancer is hindered by the perinephric fat and the position of the kidneys behind the ribs; however, as the technology improves with image fusion, faster treatments, and the ability with phased array transducers and motion compensation to overcome the problems caused by the ribs and breathing, successful treatment of kidney tumours will become more of a reality. In due course, there will be a new generation of machines for treating prostate cancer. These devices will further minimise the side effects of radical treatment of prostate cancer.

## 1. Introduction

High-intensity focused ultrasound (HIFU) has been a topic of interest for medical research for 80 years. In contrast to diagnostic ultrasound, where frequencies range from 2–15 MHz with an intensity of 0.1 watt per cm^2^, high-power applications of ultrasound often use frequencies between 300 kHz and a few MHz, with focal intensities that can be as high as 1500 watts cm^−2^. However, if the ultrasound beam carries sufficient energy and is brought to a tight focus, the energy concentration in the focal region can cause a local rise in temperature that is high enough to cause thermal tissue necrosis (a ‘lesion’). Temperatures in excess of 56 °C held for a few seconds are required. This process is similar in principle to focusing the sun’s rays through a magnifying glass onto a point, although unlike light, ultrasound does not propagate through air. A focused ultrasound beam can pass into the body without damage to surrounding tissues overlying the focal region, provided it is coupled to the skin in a way that excludes any air pockets (using, for example, water, gel, or a light oil). Its ability to cause cell death in a volume of tissue distant from the ultrasound source makes HIFU an attractive option for development as a non-invasive surgical tool.

Ultrasound causes tissue damage through two mechanisms: heat and cavitation. As an ultrasound beam propagates through tissue, some of its energy is deposited as heat, but in diagnostic fields this heat will dissipate rapidly. When the rate of heating exceeds the rate of cooling, the result will be a local temperature rise. Above a threshold of a one second exposure at 56 °C, thermal toxicity occurs, leading to irreversible cell death through coagulative necrosis. In the context of HIFU, the temperature at the focus can rise rapidly above 80 °C [1], which will lead to effective cell killing even at very short exposures [2]. At temperatures above 43 °C, the time to achieve necrosis halves with every degree rise in temperature. 

There is a steep temperature gradient between the focus tissue and neighbouring tissue, which results in a sharp demarcation on histology between the lesion and normal surrounding cells. The cooling effect of perfusion, which limits the reproducibility of other forms of hyperthermia treatment, can be practically eliminated by keeping exposure times below three seconds [3]. Acoustic cavitation—the activity of micron-sized gas bubbles under the action of an ultrasound field—is complex and unpredictable, but the end result is also cell necrosis, caused by a combination of mechanical stresses and thermal injury. Ultrasound causes the tissues to vibrate and the molecular structure is subjected to alternating compression and rarefaction. During rarefaction, gas can be drawn out of solution to form bubbles, which oscillate in size or collapse rapidly, causing mechanical stresses and generating temperatures of up to 2000–5000 ^o^C in the microenvironment [4]. Among other factors, the probability of cavitation occurrence is dependent on pulse length, frequency, and intensity [5]; while unlikely to occur with diagnostic ultrasound, cavitation may be an important consideration when using HIFU. Bio-effects from tissue heating are both more repeatable and more predictable than cavitation [6], making tissue heating the preferred mode of action in most clinical applications of HIFU, although mechanical disruption through cavitation may enhance the development of antitumour antibodies, which could lead to a beneficial effect on metastatic disease [7]. Non-thermal cavitation-mediated tissue emulsification is now being used to treat renal and prostate cancers in a technique known as histotripsy [8].

## 2. History of HIFU

The history of medical ultrasound goes back more than a century to 1880, when Jacques and Pierre Curie reported the phenomenon of piezoelectricity [9]. In 1920, Langevin demonstrated the potential of piezoelectric materials as sources of ultrasound [10]. In 1927, Wood and Loomis first described the biological effects of high-intensity ultrasound [11]. In 1942, Lynn et al. [12] published the first paper highlighting some of the possible therapeutic applications of ultrasound, and in the next decade, William Fry was able to produce HIFU lesions deep in the brains of cats and monkeys [13,14]. His brother Frank subsequently treated patients with Parkinson’s disease and other neurological conditions [15]. However, in the early days of brain HIFU, it was necessary to perform a craniotomy before treatment. Another early application for HIFU was in the treatment of glaucoma. Unfortunately for the HIFU field, lasers were also being developed at this time, and their perceived easier use led to their preferred uptake. The first suggestion that HIFU could be used for treating cancer came from Burov in 1956 [16], and in the following years several, studies examined the effects of ultrasound on tissues [17]. The specific properties of focused ultrasound propagation and its modes of destruction in normal tissues were investigated further during the 1970s and 1980s [18,19,20]. Studies using HIFU to treat experimental tumours followed [21,22].

Today, there are a number of HIFU devices in general use, both extracorporeal for abdominal applications and, more recently, for use in the brain and in intracavitary applications (most commonly transrectal for the treatment of prostate cancer). Although the components of the delivery systems are similar, treatments can be guided and monitored using ultrasound or magnetic resonance imaging. Increasingly, these modes are being fused together for the next generation of machines.

In general, the ultrasound-guided (USg) devices are smaller and more flexible than devices using magnetic resonance imaging (MRg). The combination of imaging and therapy sources into one treatment head allows a wide range of mounting designs, such as devices used in transrectal probes or on robotic arms. The extracorporeal USg device that has been used most frequently to date has this combined head mounted in a water bath below a treatment bed, in a similar geometry to that of MRg devices. Successful ablation is seen on ultrasound images as newly created bright echoes in the target volume. These echoes are due to the production of bubbles, usually resulting from tissue water boiling. MRg-guided systems arguably provide more readily interpretable images than ultrasound, and as temperature-sensitive imaging sequences are available, thermal maps can be produced during treatment. However, these systems are dependent on clinical MRg scanners, which in some settings deny their use for diagnostic imaging purposes.

With the exception of the transurethral ultrasound ablation (TULSA) system for the treatment of prostate cancer, intracavitary devices use ultrasound guidance. Table 1 summarises intracavitary devices that are currently available for clinical use.

## 3. Urological Applications of HIFU

Currently, the main urological application of HIFU is for the treatment of prostate cancer; although, in the 1990s, several groups investigated the potential of HIFU for the treatment of benign prostatic hyperplasia. While all investigators saw some improvement in symptom scores and flow rates, many needed further interventions and HIFU dropped out of the therapeutic armamentarium. These studies were reviewed by Kennedy et al. [23]. Early studies of bladder cancer had a similar fate. Preclinical work led to a feasibility study by Watkin et al. in pigs [24] and a Phase II study by Vallancian of 25 patients with a single superficial bladder recurrence [25] that showed 67% tumour-free results at 1 year. However, the technology was much more complicated than cystoscopy and still required general anaesthesia, so fell into disuse.

### 3.1. Kidney

There have been a number of experimental studies in which benign and malignant tissues within the kidney have been destroyed by HIFU [26,27], but in all of these preliminary studies there were problems with skin damage and a wide variation in the extent of tissue ablation.

A number of Phase 1b and 2a trials have been carried out in Oxford, considering HIFU for the treatment of renal tumours using a USg system [28,29,30]. Initially, eight patients with renal tumours were treated. A subsequent study investigated the use of HIFU for the management of small renal tumours over a three-year period. This trial was comprised of 17 patients with an initial radiological diagnosis of renal malignancy and with a mean tumour size of 2.5 cm.

Fourteen patients were available for evaluation at six months, and eight tumours had involuted. Four patients had other treatment due to irregular contrast enhancement on subsequent imaging, suggesting incomplete ablation of the tumour, while the other patients maintained loss of enhancement and a mean decrease of 30% in the tumour area. This study showed that HIFU achieved stable lesions in two-thirds of patients, with minimal morbidity. A further study was then carried out, wherein patients with renal tumours smaller than 4 cm were initially treated with HIFU prior to surgical resection by a partial nephrectomy, and the subsequent specimens were examined histologically. This study showed similar findings to the previous study with variable degrees of ablation, but the findings were complicated by the amount of subcutaneous and perinephric fat that absorbed much of the energy and by the position of the tumour in relation to the ribs. To investigate the first of these problems, ten patients undergoing renal cancer surgery had the perinephric fat examined separately to see the percentage output drop in energy as the thickness of the fat increased. The attenuation was significant, decreasing intensity from 58% at 2 cm to 26% at 5 cm [31]. Furthermore, it has been shown that the fat surrounding the kidney is different in females than in males, in whom it is more compact [32]. This difference could affect the planning of HIFU treatment for kidney cancer in female patients. 

High acoustic outputs are needed to compensate for this intensity loss, which in turn leads to an increased risk of pre-focal and surrounding tissue damage and subsequent loss of image quality due to pre-focal swelling. The problem of ribs and perinephric fat, which complicates HIFU in a native kidney, disappears for a renal transplant that has had its fat removed and placed in the iliac fossa. Two renal transplant patients with tumours in their transplants have been treated with HIFU in Oxford. The first treatment was unsuccessful, with the patient suffering skin burns due to technical problems from malpositioning of the water balloon inserted between the transducer and the patient to aid the targeting of the HIFU focus. The second treatment [33] had a good technical result with lack of contrast uptake on the gadolinium-enhanced MRI scan. A further biopsy of the lesion was taken and further malignant cells were seen, so a partial nephrectomy was performed to preserve renal function. Histological review of the specimen demonstrated 90% ablation of the tumour with a small rim of viable tumour cells still present.

A laparoscopic HIFU system (Sonatherm, Misonix Inc, Farmingdale, NY, USA) has also been developed for the treatment of small renal masses. The first in-human treatment was performed at the Churchill Hospital in Oxford in 2006. Phase 1 studies for the treatment of small renal tumours using this device were performed in Oxford and Vienna [34]. For 10 patients in Vienna and 12 patients in Oxford, these studies showed that laparoscopic HIFU was feasible and safe for the treatment of small renal tumours with good ablation and low morbidity, but laparoscopic HIFU is invasive in contrast to non-invasive extracorporeal HIFU. The authors concluded that the technique needed further evaluation in Phase II studies. Due to financial constraints, no further studies were performed.

Other studies have shown a gender-related distinct metabolic pattern for approximately 89% of genes, several of which encode for proteins that have a role in important biological pathways. It has been shown that RCCs from male patients overexpress genes involved in immune response and inflammation, while whole RCCs from female patients overexpress genes related to the catabolic process [32]. This distinction suggests a potentially different therapeutic approach in females who are able to exploit this metabolic switch. While this consideration may not affect the ablative aspects of HIFU, it could affect cavitational aspects that are more likely to induce an immune response. In this sense, HIFU could have a more effective role in females with small RCC masses, as compared to men. Future trials bases on these findings could be very promising.

### 3.2. Prostate Cancer

Over the last two decades, HIFU has been expanding its indications, from its early days in patients with localised disease unfit for more radical treatment to partial ablations for focal disease, and to salvage therapy following primary surgery and radiotherapy [35]. At the time of writing, prostate cancer treatment using transrectal HIFU under general anaesthesia has shown promise in more than 100 sites across the world [36].

Survival rates are not good outcome measures for assessing prostate cancer treatments, as has been demonstrated in the ProtecT study [37,38], in which the 10-year median follow-up outcomes after monitoring active surveillance, surgery, or radiotherapy for localised prostate cancer, with over 500 patients randomised to each group, showed the same 99% 10-year disease-specific survival. Complications vary depending on the series; although, in 2019, patient-reported outcomes for radical surgery and radiotherapy for prostate carcinoma were published by the National Prostate Cancer Audit, which received replies from over 25,000 men who had treatment between April 2015 and September 2016. In these two years of treatment for localised disease, 1 in 10 men experienced what was described as a severe urinary complication following surgery or a severe bowel complication following external beam radiation. On a scale of 1 (best) to 100 (worst), sexual function post-surgery was 23 and urinary continence was 71. After radiotherapy the figures were 17 and 85, respectively.

Although the apparent incidence of prostate cancer has increased over the years, mainly as a result of PSA screening, this method continues to be controversial [38,39]. However, with this improving diagnosis and the search for less invasive treatments, HIFU has provided another option in the armamentarium. Madersbacher et al. first reported in 1995 that HIFU could destroy prostate carcinoma, [40] and since that time there have been many studies, mainly using two commercially available therapy systems, the Ablatherm^®^ (EDAP-Technomed, Lyon, France) and the Sonablate^®^500 (Focus Surgery, Indianapolis, IN, USA), which use endorectal probes containing both the therapy transducer and ultrasound imager.

Ahmed et al. reported the first UK series of HIFU treatments for prostatic carcinoma, reporting a significant drop in prostate-specific antigen [41]. In 2017, the French Urological Association reported results of a six-year prospective IDEAL [42] multi-institutional study (2009–2015), which evaluated HIFU-hemi-ablation as a primary treatment in 111 patients with medium- or low-grade unilateral localised prostate cancer. The report found that at one year there was a 95% absence of clinically significant cancer, and at two years there was a treatment-free survival rate of 89% [43].

Dickinson et al. [44] reported medium-term outcomes in 569 men from a national multi-centre registry cohort receiving primary whole-gland HIFU (Sonablate 500) for primary non-metastatic prostate cancer. Redo-HIFU was permitted as part of the intervention, leading to a total of 754 interventions. They found that whole-gland HIFU is a repeatable day-case treatment that confers low rates of urinary incontinence: 12% of the patients, who had not previously required pads for incontinence, now required them. Disease control at a median of just under five years of follow-up demonstrated the potential of whole-gland HIFU as a treatment for non-metastatic prostate cancer. Endoscopic interventions and erectile dysfunction rates were similar to those found with other whole-gland treatments, with 61% having erectile problems post-HIFU. Only one patient developed a recto-urethral fistula (0.13%).

Stabile et al. [45] reported on the medium-term oncological outcomes following focal treatment using the Sonablate 500 system in 1032 men, 80% of whom had a Gleason score of 3 + 4 or above. While the overall survival rate of 97% at 96 months is not surprising, they found that freedom from radical treatment was 81% at 96 months and concluded that focal HIFU for prostatic carcinoma is a feasible therapeutic strategy, with acceptable survival and oncological results and a reduction in the five-year retreatment rates.

Similarly, Abreu et al. recently reported the first series of focal HIFU for prostate cancer in 100 men in the United States. Although retrospective in nature, the report did show good results, similar to other studies, in terms of disease control and genitourinary function, with 91% of patients avoiding radical treatment and all patients maintaining full continence after two years with no major adverse events [46].

HIFU treatment has been used in a number of studies for salvage treatment. In a study limited by its retrospective nature, Crouzet S et al. [47] looked at the oncological outcomes from 1995-2009 in 418 patients of salvage high-intensity focused ultrasound for locally recurrent prostate cancer after external beam radiotherapy. The study found a 7-year cancer-specific survival of over 80%, but at the price of significant morbidity. However, complication rates of incontinence, outflow obstruction, and recto-urethral fistula decreased significantly after 2002, when treatment-specific parameters for post-radiotherapy salvage HIFU were introduced to account for the decreased vascularisation of the prostate gland and peri-prostatic tissue that resulted from radiotherapy-induced fibrosis. Using these amended parameters, the recto-urethral fistula rate decreased from 9% to 0.6%.

Similarly, Houstiou et al. [48] evaluated the oncological and functional outcomes of salvage high-intensity focused ultrasound for locally recurrent prostate cancer after low-dose-rate brachytherapy in a clinical Phase II study of 50 patients between 2003 and 2015. They found satisfactory disease control, and the complication rate was decreased with specific post-brachytherapy parameters of the HIFU delivery and hemi-ablation when possible. Kanthabalan et al. [49] found a similar benefit of focal salvage therapy in a study of 150 men.

A few units have looked at transurethral ultrasound ablation of the prostate (TULSA). Bonekamp et al. [50] demonstrated a median 12-month reduction in viable prostatic volume of 88% in 29 patients in a radiology review of the Phase I clinical trial of MRI-guided transurethral ultrasound ablation (TULSA) in patients with localised prostate cancer.

Although no large randomised clinical trials of HIFU in prostate cancer have been conducted, the ‘Partial ablation versus radical prostatectomy in intermediate-risk prostate cancer: the PART feasibility RCT study’ [51] demonstrated that randomisation of men to an RCT comparing partial ablation with radical treatments of the prostate is feasible. The full study has received funding from the NIHR and was scheduled to start in autumn 2020.

## 4. Conclusions

Sir William Osler, who ended his career as the Regius Professor of Medicine in Oxford and died in 1919, is reputed to have said that “diseases which harm require treatments that harm less”. Technology does not go backwards. There is no doubt that HIFU works and is now an accepted treatment for localised prostate cancer, but not yet for renal cancer due to the absorption of energy by the perinephric fat and the position of the kidneys behind the ribs. However, as the technology improves with image fusion, faster treatments, and the ability with phased array transducers and motion compensation to overcome the problems caused by the ribs and breathing, successful treatment of kidney tumours will become more of a reality. In due course, there will be a new generation of machines for treating prostate cancer. These devices will further minimise the side effects of radical treatment of prostate cancer, and with further research and greater understanding of the genetics of prostate cancer, the right people will be treated in the right way at the right time and for the right reason. As the world faces up to the challenges presented by the coronavirus pandemic, successful non-invasive surgical treatments will become increasingly important in the next decade.

## Figures and Tables

**Table 1:** Intracavitary devices being used for the treatment of prostate cancer.

Company	Device	Treatment Frequency Range	Reach	Guidance Method	Power Settings	
Edap Technomed	Focal One (formerly Ablatherm)	3 MHz	32–67 mm ^1^	US 7.5 MHz ^2^ US/MR fusion	Set levels, automatically calculated depending on focal length & area to be treated.	Trans-rectal
Sonacare Medical	Sonablate	4 MHz	3 cm & 4 cm focal lengths ^3^	US 6.5 MHz ^4^ US/MR fusion	Adjustable ^5^	Trans-rectal
Profound Medical	TULSA-PRO	4.1–4.5 MHz 13.0–14.4 MHz	3 cm ^6^	MRI (1.5 T or 3 T)	Automatically set and continuously adjusted ^7^	Trans-urethral
Insightec	Exablate Prostate	2.3 ± 0.25 KHz	15–60 mm ^8^	MR (1.5 T or 3 T)	Automatically set and adjustable ^9^	Trans-rectal

Notes: ^1^ The focal length is continuously variable from 32 to 67 mm (dynamic focusing); it is automatically adjusted according to contours of the prostate or the lesion size drawn by the physician. Lesion lengths range from 5 to 45 mm. ^2^ If pre-treatment MR images are available, elastic MR fusion can automatically match the 3D contours of the prostate or lesion size on the MRI and the scanned USg images. If biopsy maps are available, biopsy map/USg fusion may be used to automatically treat the positive area. Treatment validation may be established with CEUS imaging during the HIFU session or with post-treatment MRI. ^3^ Sonablate operates using multiple focal zones (generally 2 or 3) based on prostate AP dimensions and location of the selected treatment tissue volume. ^4^ Imaging Modes: transverse and sagittal planes simultaneously; 3D rendering of the prostate allows the user to image prostate and surrounding tissues in transverse, sagittal, and coronal planes. Treatment planning: selection of any volume and shape by the user. Tissue Change Monitoring (TCM): real-time calculations of changes of tissue as a function of temperature based on backscattered signals changes. ^5^ The treatment has default power values (37 W and 24 W for 4.0 and 3.0 cm focal lengths respectively) for treating selected tissue volume; however, the power is adjusted if the tissue is not located exactly in the focal zones. Moreover, power is adjusted by the user to create visual feedback (“popcorn”) and adjusted to correct variability and changes of ultrasonic attenuation due to temperature. ^6^ Measured radially from urethra. ^7^ Power is controlled by software in a closed feedback loop using MR thermometry. ^8^ Variable focal distance is adjusted and controlled automatically by the system based on a physician-generated treatment plan. Achieved by electronic steering with the 990 transmit elements flat phased array transducer. ^9^ Acoustic power set to 30 W acoustic by default in order to avoid cavitation effects. Maximum acoustic power allowed: 45 W acoustic. User is able to adjust power level to adjust ablation based on measured tissue response. T stands for tesla.

## Data Availability

Pub med was used to search articles but nothing else used.
